# Transcriptome profiling reveals transcriptional and alternative splicing regulation in the early embryonic development of hair follicles in the cashmere goat

**DOI:** 10.1038/s41598-019-54315-7

**Published:** 2019-11-28

**Authors:** Yanjun Zhang, Lele Wang, Zhen Li, Dong Chen, Wenjing Han, Zhihong Wu, Fangzheng Shang, Erhan Hai, Yaxun Wei, Rui Su, Zhihong Liu, Ruijun Wang, Zhiying Wang, Yanhong Zhao, Zhixin Wang, Yi Zhang, Jinquan Li

**Affiliations:** 1 College of Animal Science, Inner Mongolia Agricultural University, 010018 Hohhot, Inner Mongolia Autonomous Region China; 2 Ulanqab Medical College, 010020 Ulanqab, Inner Mongolia Autonomous Region China; 3 Center for Genome Analysis, ABLife Inc., Wuhan, Hubei 430072 China; 4 Key Laboratory of Mutton Sheep Genetics and Breeding, Ministry of Agriculture, 010018 Hohhot, Inner Mongolia Autonomous Region China; 5 Key Laboratory of Animal Genetics, Breeding and Reproduction in Inner Mongolia Autonomous Region, 010018 Hohhot, Inner Mongolia Autonomous Region China; 6 Engineering Research Center for Goat Genetics and Breeding, Inner Mongolia Autonomous Region, 010018 Hohhot, Inner Mongolia Autonomous Region China

**Keywords:** Differentiation, Transcriptional regulatory elements

## Abstract

The undercoat fiber of the cashmere goat, from the secondary hair follicle (HF), possesses commercial value. However, very few studies have focused on the molecular details of primary and secondary HF initiation and development in goat embryos. In this study, skin samples at embryonic day 45, 55, and 65 (E45, E55, and E65) were collected and prepared for RNA sequencing (RNA-seq). We found that the HF probably initiated from E55 to E65 by analyzing the functional pathways of differentially expressed genes (DEGs). Most key genes in canonical signaling pathways, including WNT, TGF-β, FGF, Hedgehog, NOTCH, and other factors showed clear expression changes from E55 to E65. We, for the first time, explored alternative splicing (AS) alterations, which showed distinct patterns among these three stages. Functional pathways of AS-regulated genes showed connections to HF development. By comparing the published RNA-seq samples from the E60, E120, and newborn (NB) stages, we found the majority of WNT/β-catenin signaling genes were important in the initiation of HF development, while other factors including *FOXN1*, *GATA3*, and *DLX3* may have a consistent influence on HF development. Our investigation supported the time points of embryonic HF initiation and identified genes that have potential functions of embryonic HF initiation and development. We further explored the potential regulatory roles of AS in HF initiation, which extended our knowledge about the molecular mechanisms of HF development.

## Introduction

The cashmere goat is well-known for producing high quality cashmere wool. The Inner Mongolia cashmere goat is a traditional cashmere goat breed in China. Previous research has shown that the fleece of cashmere goats consists of two distinct fibers: guard hair and cashmere undercoat, which are produced by primary hair follicles (PHF) and secondary hair follicles (SHF), respectively^1^. The undercoat is fine and soft, while guard hair is straighter and much coarser than the undercoat. The undercoat of the cashmere goat is the source of cashmere products such as pashmina or sweaters, implying its high commercial value^1–4^. Hair follicle (HF) organogenesis is generated during fetal skin development and relies on ectodermal-mesodermal interactions^5,6^. During cashmere goat embryonic development, the hair placodes of PHF are initiated at 45–55 days gestation. The formation of SHF is initiated at 65–75 days gestation, and all PHFs and some SHFs mature at 135 days gestation^7^. These two kinds of HFs present different structures^7^. As a consequence, we propose that understanding the molecular features and underlying regulatory mechanisms that define the embryonic precursors in nascent cashmere follicles may help produce high yielding cashmere goats.

There have been several studies focusing on the critical molecular mechanisms of HF development in the past decades^8–10^. In the past 20 years, many studies have focused on the molecular mechanisms of HF development and cycling control in humans and mice^5,11–13^. Increasing evidence shows that HF transformation during cycling is caused by alterations in the local signaling milieu^5,6,13,14^. Previous studies in humans and mice also demonstrated that several pathways like WNT/beta-catenin, TGF-beta/BMP, FGF, Hedgehog, NOTCH, and other factors (SOX9, LHX2, DLX3, GATA3, RUNX1, TP63, MSX2, FOXN1, NFATC1, and TBX1) play important roles in HF development^8^. However, among these studies on HFs, few have been carried out in cashmere goats, whose skin is comprised of two types of HF, and studies are also lacking on the early stages of HF development^15,16^. The molecular mechanisms of PHF initiation that occur at early stages in embryonic development, and the transcriptional gene expression and alternative splicing related to HF morphogenesis in cashmere goats, still remain to be elucidated. A better understanding of the biological characteristics and regulation of HF morphogenesis may provide approaches to enhance the formation of fleece and achieve higher fleece production.

Nowadays, high-throughput RNA sequencing (RNA-seq) allows a rapid and comprehensive examination of the transcribed genomic elements in the genome and helps researchers discover novel protein-coding and non-coding transcripts, showing more powerful capabilities compared with traditional methods based on public sequence information^17^. Several studies have been performed to investigate transcriptome profiling changes during HF development using RNA-seq technology^18–21^. Seasonal HF cycling differences between cashmere and milk goats showed several key factors for HF cycling^22^. Embryonic samples taken from day 60 (E60), 120 (E120), and newborn (NB), which correspond to SHF initiation, PHF, and SHF maturation, respectively, were sequenced to explore HF development in cashmere goat embryos^23^. Many publications have explored fetal HF development and individual cycling in mice^24–26^, while the molecular mechanisms of HF initiation and development in cashmere goats needs to be further illuminated.

In order to enhance our understanding of the process of cashmere goat fetal HF initiation, we used RNA-seq and bioinformatics analysis to study the PHF and SHF development in cashmere goat embryos. Skin epidermis samples were taken at early stages of cashmere goat fetal HF development. We assayed the gene expression profiles at these initial stages of HF morphogenesis and explored the potential molecular mechanisms by which signaling cascades in embryonic skin epidermis regulate HF formation. Meanwhile, the impact of alternative splicing (AS) on HF development was investigated for the first time. We found that the AS profile was distinct among the three different embryonic stages, implying potential regulatory roles in HF development.

## Results

### Transcriptome profiling of gene expression dynamics during early hair follicle development

To determine the transcriptional profiles at the initial stages of embryonic HF development in cashmere goats, we selected skin samples at E45, E55, and E65. According to our previous observation, the formation PHF and SHF initiate at E55-E65 and E65-E75, respectively^7^. A total of nine RNA-seq libraries (three biological replicates at each stage) were constructed. We obtained 66,882,470 to 95,111,216 raw reads for each sequencing sample (Table S1). After aligning the quality filtered reads to the goat (*Capra hircus*) genome^27^, 64.19% to 81.8% of the total reads from each sample were successfully aligned. Between 39,319,085 and 71,694,863 reads per sample were uniquely aligned to the genome. Overall, 22,987 of the 28,921 annotated genes (79.48%) were detected in these samples. We used RNASeqPowerCalculator^28^ to calculate the statistical power (Fig. S1) and other parameters (Table S2) for our RNA-seq data.

**Figure 1:**
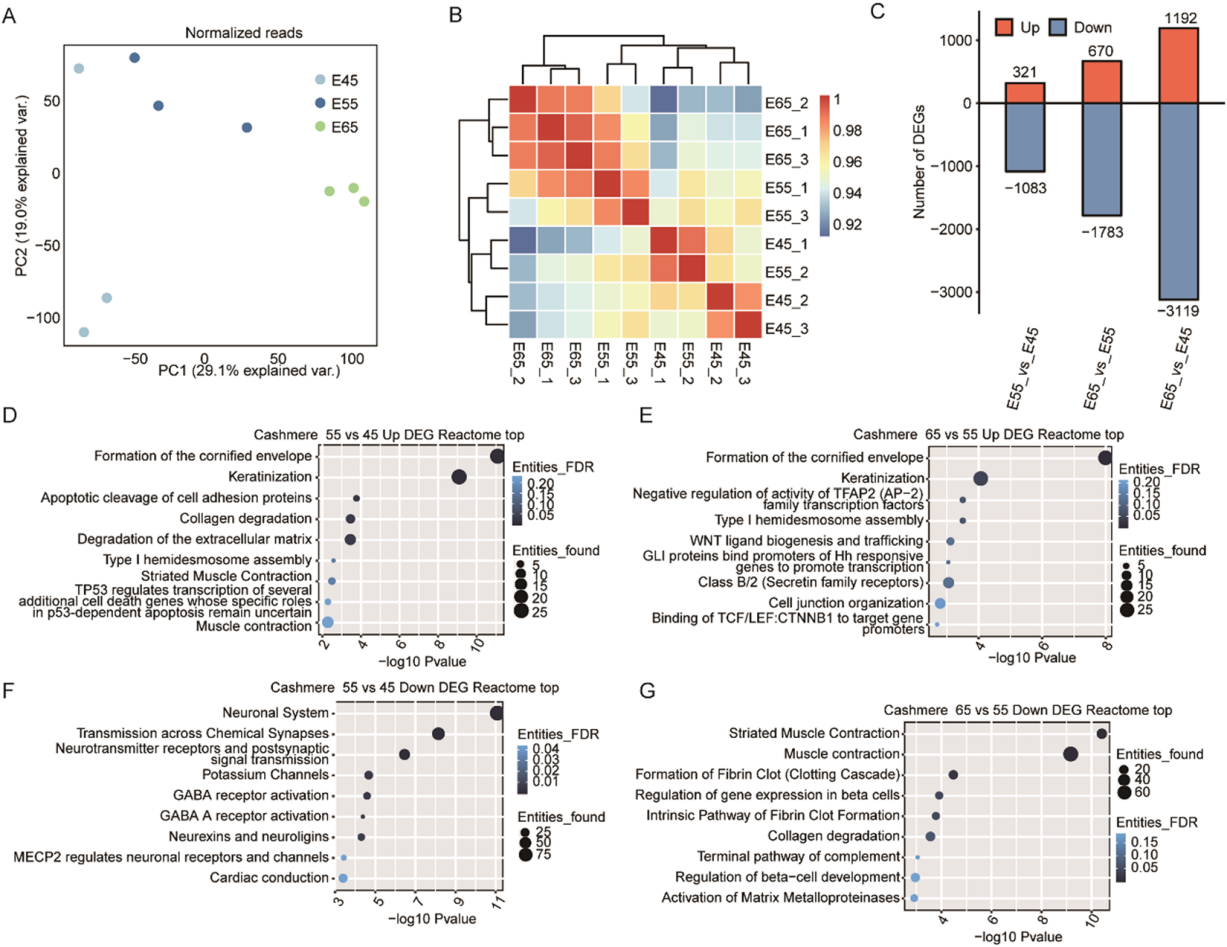
The global transcriptional profiles and the enriched functional analysis of DEGs. (**A**) Principal component analysis (PCA) of nine samples based on normalized mRNA expression level. The samples were grouped by different embryonic days. (**B**) Heat map showing the clustering of the nine embryonic samples by calculating the Pearson’s correlation coefficients using expression values. (**C**) Bar plot showing the number of differentially expressed genes in three comparison groups (E55 vs. E45, E65 vs. E55, E65 vs. E45). Red bar represents the number of upregulated genes while blue represents the number of downregulated genes. (**D–G**) Bubble plot showing the top enriched Reactome pathways of the DEGs.

Principal component analysis (PCA) was performed to cluster the nine samples from the three stages based on gene expression level. PC1 showed that the distance between the E45 and E55 samples is less than that between E55 and E65. The three E65 samples were close to each other but distinct from the E45 and E55 samples. However, the E45 and E55 samples were more discrete than the E65 samples (Fig. 1A). Sample correlation analysis of the nine samples confirmed the results from PCA (Fig. 1B). To explore the transcriptional differences among these stages, we identified a total of 4,962 differentially expressed genes (DEGs) using the edgeR package^29^ with a false discovery rate (FDR) < 0.05 and|log_2_ fold change| ≥ 1. Among them, 1,083, 1,783, and 3,119 were downregulated and 321, 670, and 1,182 DEGs were upregulated in the E55 vs. E45, E65 vs. E55, and E65 vs. E45 groups, respectively (Fig. 1C). These results showed that more downregulated genes were detected than upregulated genes at the initial stages of HF development, and the transcriptional difference between E55 and E65, during which time PHF is initiated, was more pronounced than that between E45 and E55.

### Transcriptional activation of genes in a number of HF developmental pathways occur before and during PHF formation

To further understand how the functions of DEGs in E65 vs. E55 and E55 vs. E45 are related to embryonic HF development, we performed Reactome analysis. For the upregulated DEGs in E55 vs. E45, the top 10 enriched pathways contained well-known pathways involved in HF development, including cornified envelope, keratinization, apoptotic cleavage of cell adhesion proteins^30^, collagen degradation^31^, degradation of the extracellular matrix^32^, and pathways related to TP53^33^ (Fig. 1D). Two muscle contraction functional terms were also included. These results probably suggested that the gene expression patterns were shifted to support HF development from E45 to E55 when the epidermal structure developed and the phenotypic appearance of the HF structure emerged. For the upregulated DEGs in E65 vs. E55, the top 10 enriched pathways revealed the presence of two HF pathways (Fig. 1E), including formation of the cornified envelope and keratinization, which were also activated during development from E45 to E55. This finding is consistent with the hypothesis that genes in these functional pathways were further activated during PHF formation. Additionally, a number of new functional pathways regulating HF formation were also activated, including the negative regulation of the TFAP2 (AP-2) family of transcription factors^24^, WNT ligand biogenesis and trafficking^8^, GLI protein binding to promoters of Hh responsive genes to promote transcription^34^, and binding of TCF/LEF:CTNNB1 to target gene promoters^35^ (Fig. 1E).

Among the top 10 enriched pathways among the downregulated DEGs in E55 vs. E45, a number of neuronal development related pathways were identified, including neuronal system, neurexins^36^, and neuroligins^37^, GABA receptor activation and GABA A receptor activation^38^, and pathways of MECP2^39^ (Fig. 1F). Muscle contraction related pathways were enriched in the downregulated DEGs in the E65 vs. E55 group (Fig. 1G). We also performed gene ontology (GO) enrichment analysis to explore the enriched functional terms for the DEGs (Fig. S2). Skin barrier and HF development related terms were also enriched in the upregulated DEG sets (Fig. S2).

**Figure 2:**
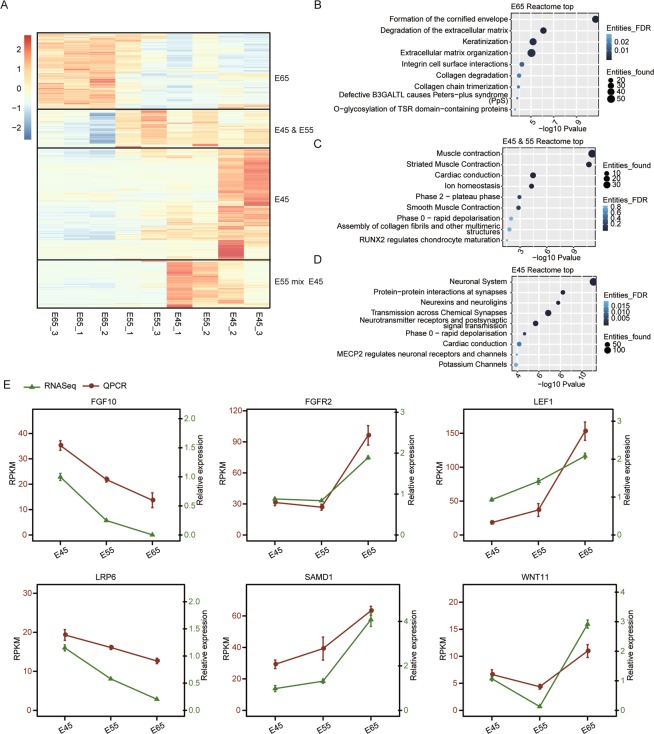
Pathway analysis of four groups clustered by heat map based on E45, E55, and E65 samples. (**A**) Heat map showing the expression pattern of all DEGs from the nine sequenced samples. The DEGs were divided into four clusters according to their distinct expression profiles. (**B–D**) Bubble plot showing the enriched Reactome pathways of the DEGs from three clusters. (**E**) Coupled line plot showing the consistency between RNA-seq and RT-qPCR quantification for the selected DEGs.

To further explore the expression dynamics of the DEGs during the initial stages of HF development, we performed hierarchical clustering analysis for all identified DEGs. Four distinct expression clusters were identified, including one E65 up-regulated and three E45/E55 up-regulated gene clusters (Fig. 2A). We then performed Reactome analysis for the DEGs from these four clusters. The activated genes at E65 were highly enriched for HF development related terms, including formation of the cornified envelope, keratinization, degradation of the extracellular matrix^32^, extracellular matrix organization^32^, collagen degradation^40^, and collagen chain trimerization^40,41^ (Fig. 2B). Interestingly, the E45/E55 upregulated gene clusters included a muscle cluster that was enriched for muscle related functional terms (muscle contraction, striated muscle contraction, and smooth muscle contraction), and HF development terms (assembly of collagen fibrils and other multimeric structures^40^ (Fig. 2C). Genes in the muscle cluster were highly expressed in both E45 and E55, and then repressed at E65. Another group of E45/E55 up-regulated genes was classified into a neuronal cluster, including neuronal system, transmission across chemical synapses, neurexins and neuroligins, MECP2 regulates neuronal receptors and channels^42^. This was a large cluster showing a high level of expression, specifically at E45 (Fig. 2D). Taken together, the DEG results provided some candidate genes that perhaps have key functions during HF initiation and development.

In addition, we conducted an RT-qPCR experiment to verify the RNA-seq results. Six DEGs from the RNA-seq analysis results were tested: *SMAD1, FGFR2, LEF1, WNT11, FGFR10*, and *LRP6*, which were involved in different signaling pathways discussed in this paper. We observed a significant increase from E55 to E65 for *SMAD1, FGFR2, LEF1*, and *WNT11*, and a significant decrease for *FGFR10 and LRP6*, showing a high agreement with the RNA-seq results (Fig. 2E).

### The expression dynamics of HF development related genes

**Figure 3:**
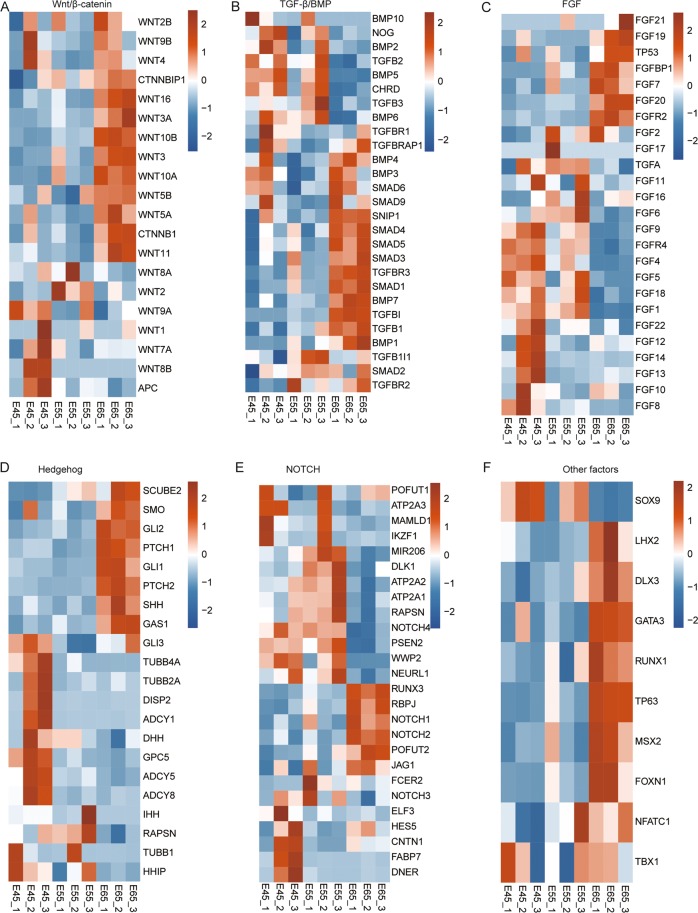
Heat map of key canonical pathway genes in hair follicle development in the E45, E55, and E65 samples (**A–F**). Genes from defined signaling pathways are developmentally regulated in embryonic skin. Published key genes for specific signaling pathways during HF development and DEGs (E55 vs. E45 and E65 vs. E55) in these canonical signaling pathways were used as query genes. Goat genes involved in WNT/beta-catenin (**A**), TGF-beta/BMP (**B**), FGF (**C**), Hedgehog (**D**), NOTCH (**E**) signaling, and other factors (**F**) were represented in the heat map if expressed (RPKM > 0) in embryonic skin samples. In the heat map, FPKM values of each gene were replaced by log2 (FPKM), then all values in each row were normalized by a scale factor S so that the sum of the squares of the values in each row was 1.0. Colors ranged from blue to red, corresponding to low to high expression levels.

Accumulating studies have suggested that canonical signaling cascades, such as WNT/β-catenin, TGF-β/BMP, FGF, Hedgehog, NOTCH, and other factors (SOX9, LHX2, DLX3, GATA3, RUNX1, TP63, MSX2, FOXN1, NFATC1, and TBX1) play important roles in HF development^8,18^. We picked these genes from the published literature and related Reactome pathways, and then examined their expression patterns from the RNA-seq data in order to gain an updated view of the molecular regulatory network that governs the initial stage of prenatal HF formation in the goat (Fig. 3). The reported genes from each canonical signaling pathway and genes we obtained from canonical signaling pathways in the E45 vs. E45 and E65 vs. E65 Reactome analysis results were used as query genes. Genes in each signaling pathway can be arbitrarily classified into two groups: increased or decreased expression patterns from E45 to E65. A majority of genes in the WNT/β-catenin, TGF-β/BMP, and other categories showed increased expression patterns in E65, while genes from the other three signaling pathways showed the opposite pattern (Fig. 3). These results suggested different functional roles for these pathways during the initiation of HF development.

### The transcriptional shift between E60 and E120 shows significant similarity between E55-E65

**Figure 4:**
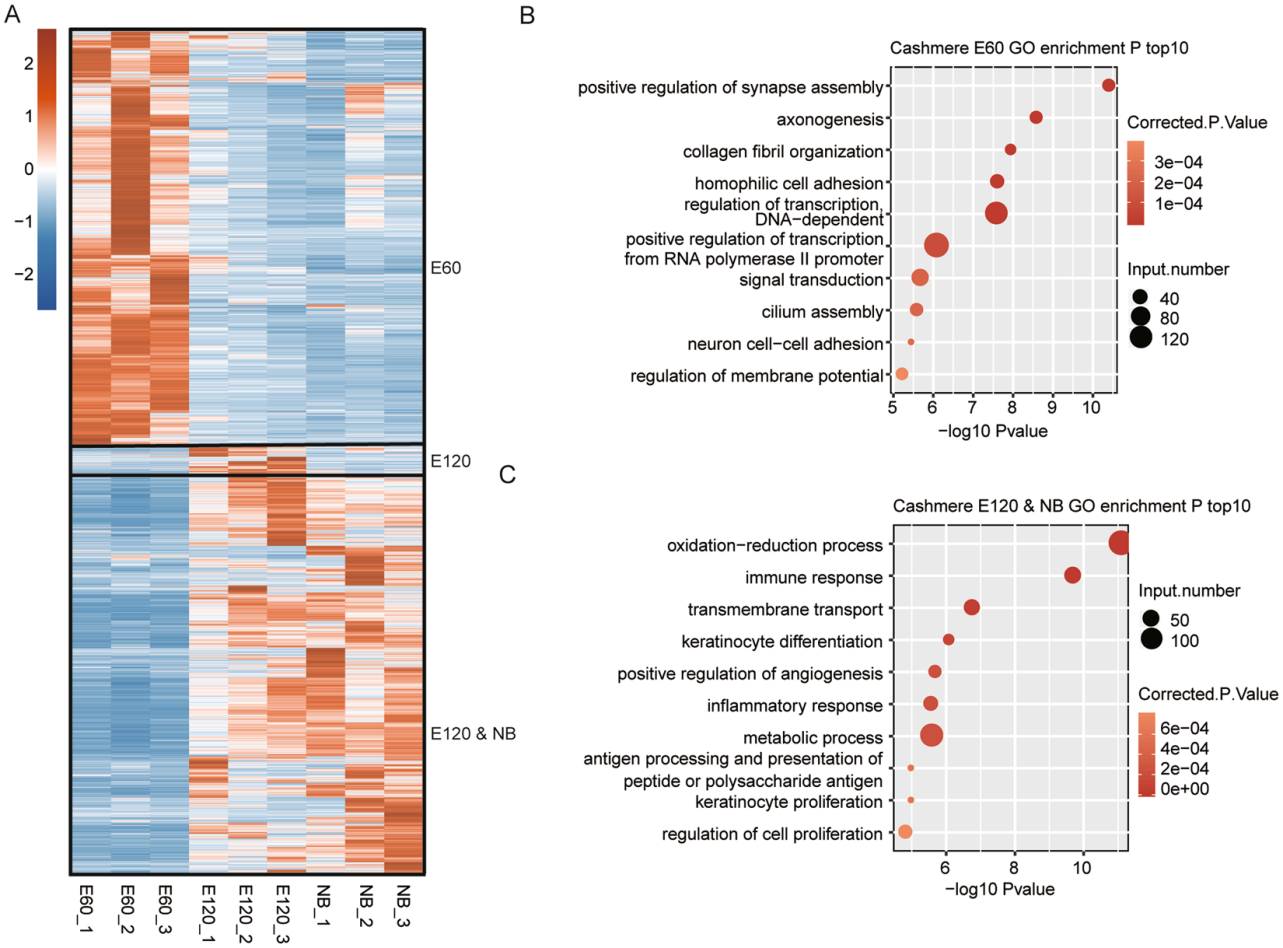
Expression patterns and functional analysis of detected DEGs based on E60, E120, and NB samples. (**A**) Heat map showing the three clusters by clustering DEGs according to their expression level. (**B–C**) Bubble plot showing the top 10 enriched GO biological process terms of DEGs in the two dominant groups.

A transcriptome study of the embryonic primary and secondary HF development in cashmere goat has been recently published^23^, including samples from cashmere goat embryos at E60 and E120, and newborns (NB). To comprehensively compare between these two datasets, we reanalyzed the RNA-seq data from that study, showing that the transcriptome profile of the E60 samples was highly distinct from those of the E120 and NB samples (Fig. S3A,B). The above results indicated that the transcriptional activation from E60 to E120 was similar to that between E55 and E65. To validate this hypothesis, we overlapped the DEGs between these two comparison groups and found the overlapped DEGs between these two DEGs were significant (*p*-value = 4.528515e-94, hypergeometric test, Fig. S3C), suggesting that thousands of genes were commonly regulated at both the early and late stages of HF development. Heat map clustering of these DEGs revealed two major clusters, the E60 and E120-NB clusters (Fig. 4A). The E120 cluster contained a very small number of genes. To explore the biological functions of genes from these clusters, GO enrichment analysis was performed. This analysis revealed that the genes from the E60 cluster were enriched in collagen fibril organization, cilium assembly, and positive regulation of synapse assembly (Fig. 4B). In contrast, the genes from the E120 and NB clusters were highly enriched in HF development related terms, including keratinocyte differentiation and proliferation, regulation of cell proliferation, transmembrane transport, and oxidation-reduction process (Fig. 4C).

**Figure 5:**
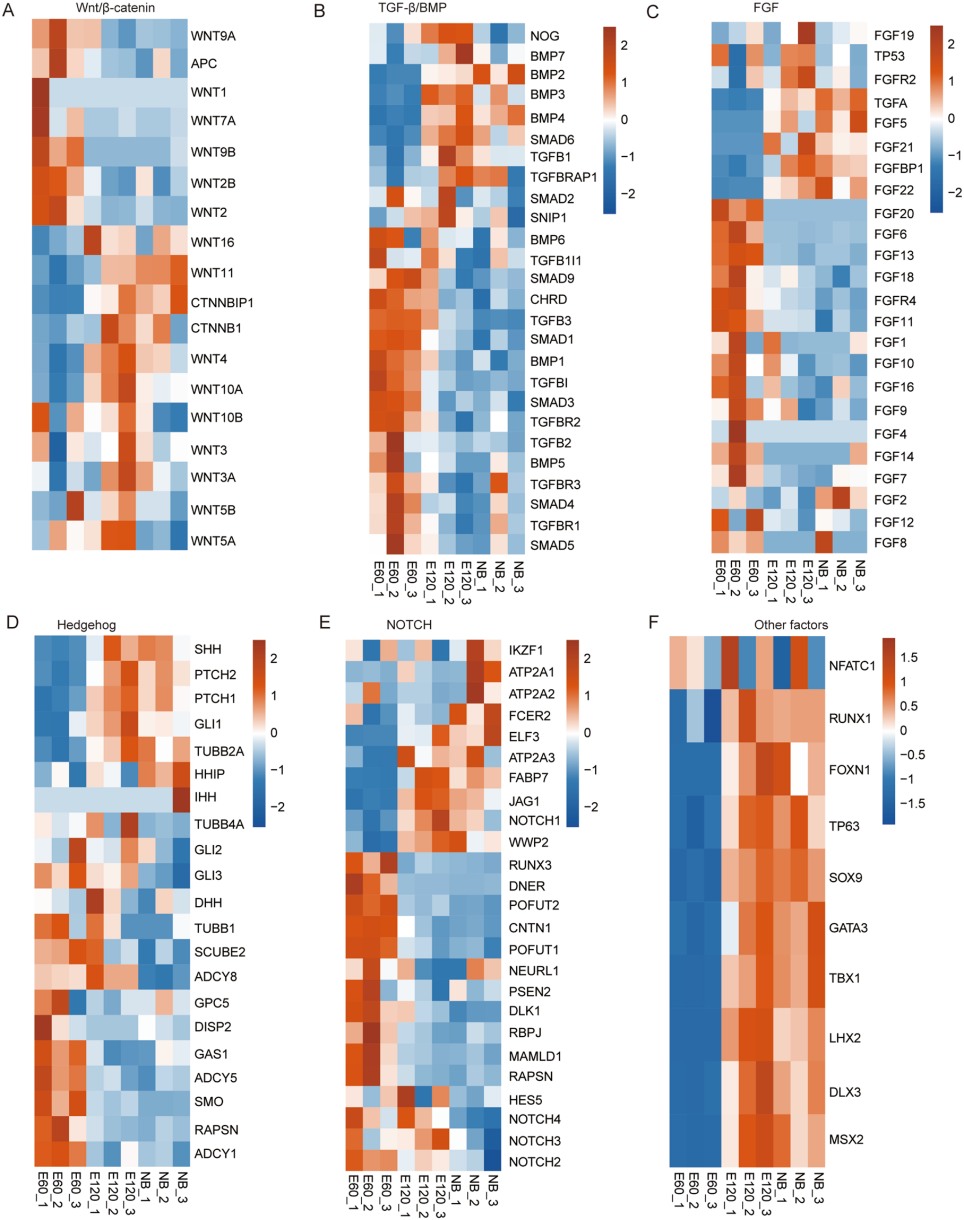
Heat map of known key genes from canonical pathways in HF development (E60, E120, and NB samples). Expressed goat genes involved in WNT/beta-catenin (**A**), TGF-beta/BMP (**B**), FGF (**C**), Hedgehog (**D**), NOTCH signaling (**E**), and other factors (**F**) are presented in the heat map.

To further explore the similarities and differences between the transcriptional switches during these stages, we plotted the expression dynamics of HF developmental genes (Fig. 5). Different from E45, E55, and E65 related to PHF initiation and development, E60, E120, and NB stages were concerned with PHF development and maturation, SHF initiation, and SHF development and maturation. We found that most genes in the WNT/β-catenin and NOTCH signaling pathways were repressed during these three stages (Fig. 5A,E), which was contrary to the expression pattern from E45 to E65. The other factors were consistently increased during these three stages (Fig. 5F).

### Alternative splicing regulation occurs during early HF development

**Figure 6:**
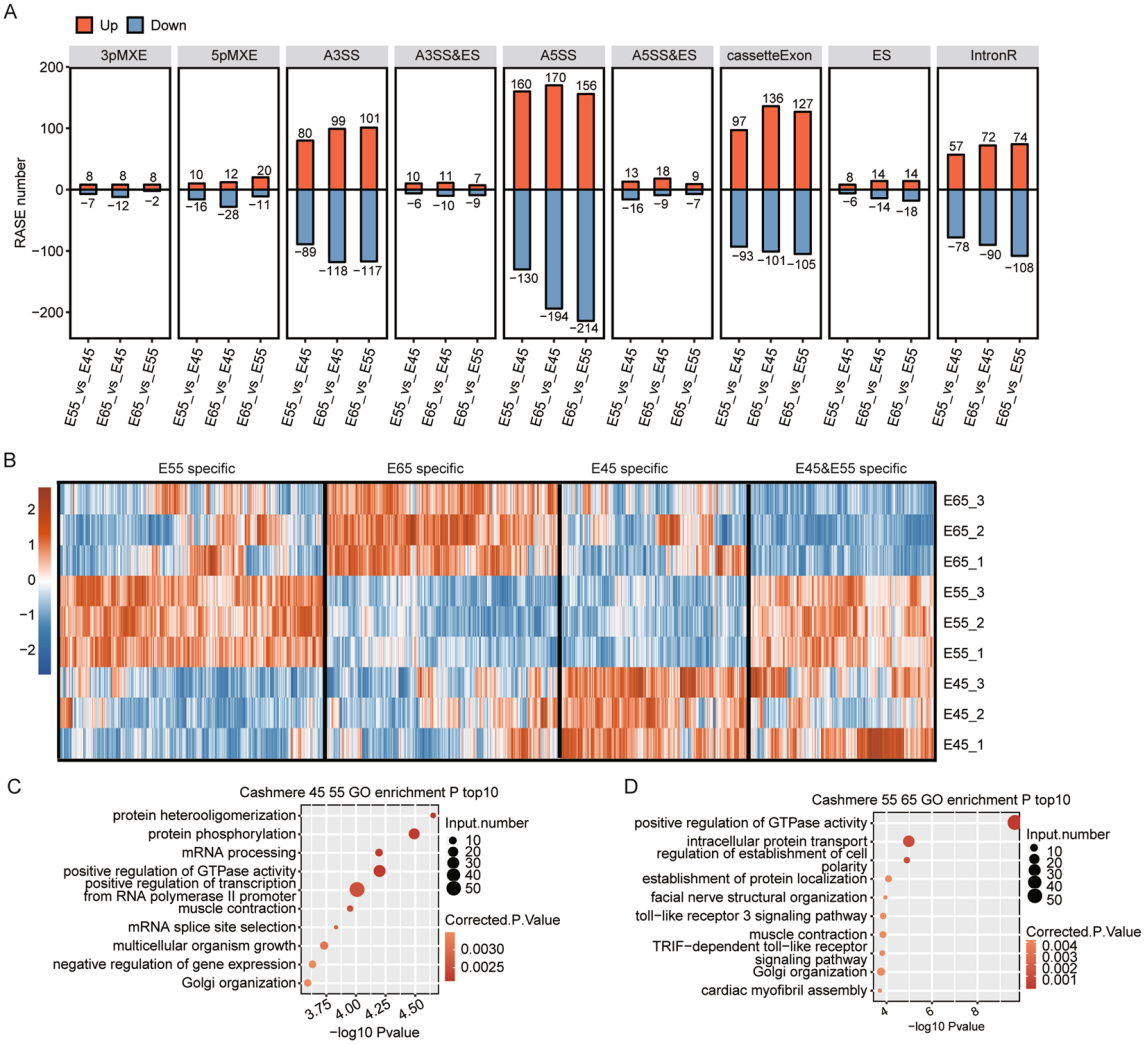
Alternative splicing analysis of E45, E55, and E65 samples. (**A**) Classification of different types of expression changes of alternative splicing events between the three stages. (**B**) Splicing patterns of different samples. A hierarchical clustering dendrogram of different AS events from the three groups. Red: relatively high ratio; blue: relatively low ratio. AS events regulated by different stages in cashmere goat embryos were divided into three groups, and the top 10 GO biological process terms of genes with RASEs in different groups were analyzed (**C–D**).

It is well-known that AS regulation is one of the key post-transcriptional mechanisms that control gene expression and protein diversity^43^. To explore the potential involvement of AS regulation in HF development, we performed AS analysis for the same samples collected from E45, E55, and E65. Regulated AS events (RASEs) during early HF development were identified (Fig. 6A). The major RASE types included alternative 5′ splice site (A5SS), alternative 3′ splice site (A3SS) and Cassette Exons. Different from transcriptional regulation, the RASEs from E55 vs E45 were only a little bit similar to those from E65 vs E55 (Fig. 6A).

We next performed heat map clustering analysis of the ratio between the alternative and model splice isoforms of all identified RASEs (Fig. 6B). Different from the dynamics of transcriptional patterns, we identified RASE clusters specific for E45, E55, and E65. Many RASEs were specific either for E55 or E45, suggesting that AS may play potential regulatory roles in HF initiation (Fig. 6B). GO functional enrichment analysis revealed that functional pathways related to protein modifications and mRNA transcription and processing emerged in genes containing RASEs from E55 vs E45 (Fig. 6C). Additionally, genes positively regulating GTPase activity were also identified (Fig. 6C). We also found that positive regulation of GTPase activity was the top pathway in the functional terms of genes containing RASEs from E65 vs E55 (Fig. 6D), suggesting that GTPase activity may be related to HF development.

**Figure 7:**
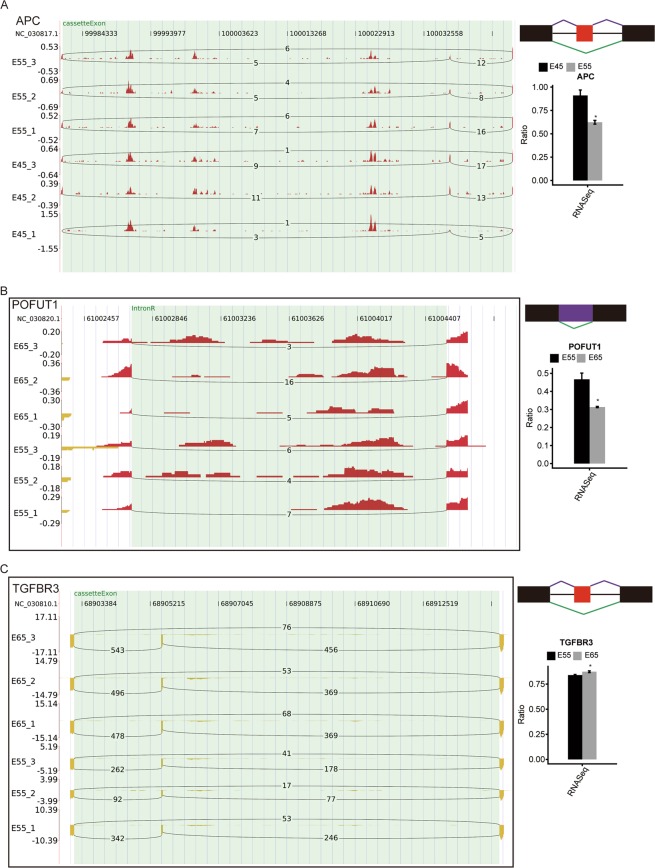
Presentation of HF development-affected RASEs. Three HF development-related genes, *APC* (**A**), *POFUT1* (**B**), and *TGFBR3* (**C**) are shown. IGV-sashimi plots show the alternative splicing changes that occurred in cashmere goat embryonic skin cells in different stages (left panel). The schematic diagrams depict the structures of alternative splicing events (right panel, top), AS1 (shown in purple), and AS2 (shown in green); exon sequences are denoted by boxes, and intron sequences by the horizontal line. RNA-seq quantification and statistical significance of RASEs are shown (right panel, bottom). The altered ratio of RASEs was calculated using the formula: AS1 junction reads/AS1junction reads + AS2 junction reads.

To illustrate the reliability of the RASE results, we selected three RASEs and corresponding genes to show their read density and statistical significance, including *APC* (exon skipping, Fig. 7A), *POFUT1* (intron retention, Fig. 7B), and *TGFBR3* (cassette exons, Fig. 7C). These three genes were among the HF development related genes^8,44^, which were also regulated by alternative splicing. In summary, these results suggested that posttranscriptional regulation, especially AS, played potential important roles in HF initiation and development.

## Discussion

Previous studies on cashmere goat embryonic HF development showed by morphological observation that PHF initiated between E55 and E65^7^. Consistent with previous studies, in our current study, samples from E45 to E55 of cashmere goat showed that most key genes, from essential pathways or important factors that influenced HF development, were upregulated in E65. We also compared the gene expression patterns between E45, E55, and E65 with the published RNA-seq data, including E60, E120, and NB samples^23^. By performing AS analysis, we found that the AS patterns of expressed genes were extensively regulated, showing that the AS profile exhibited clear differences between these three stages. Some enriched signaling pathways of AS-regulated genes were also related to HF development, suggesting that AS may participate in the initiation of PHFs.

During cashmere goat embryonic development, the complete structure of the skin epidermis was formed during E45 to E55^7^. At this stage, keratinocytes were evenly distributed in the stratum basale. The onset of PHF development occurred from E55 to E65, which is marked by the formation of a regular array of placodes, and epidermal keratinocytes were clustered in response to signals emanating from the dermis^7^. Consequently, the DEGs in E55 vs. E45 were expected to differ from those in E65 vs. E55. We found that there were 321 upregulated and 1083 downregulated genes (E55 vs. E45), and 670 upregulated and 1783 downregulated genes (E65 vs. E55). This large transcriptional shift gave us a possibiligy that HF initiation most probably occurred from E55 to E65. The dynamic expression profiles of members of the Wnt/beta-catenin family, the Hedgehog family, members of the TGF-beta/BMP pathway, the Fgf family, the Notch family, and other important factors (SOX9, LHX2, DLX3, GATA3, RUNX1, TP63, MSX2, FOXN1, NFATC1, and TBX1) further supported the above conclusion.

For example, in the Wnt/beta-catenin signaling pathway, several lines of evidence have shown that wnt signaling and beta-catenin, as well as their interactors, are critical for HF induction^10,25,45–47^. In mice, Wnt10a, Wnt10b, CTNNB1, and Lef1 are highly expressed in hair placode epithelium^48,49^. Conversely, constitutive activation of CTNNB1 induces precocious placode formation and multiple hair germ (HG) budding out from the original follicle^25,46,50^. The downstream transcription factors of wnt signaling, including LEF1, TCF3, and TCF4, have been shown to extensively regulate the development of HF^35,51–53^. From our results, the higher expression of *LEF1* and *TCF3/4* at E65 compared with E45 and E55 suggests that they play a critical role in HF initiation. Twelve genes in the wnt signaling pathway showed an increased expression level, while only seven genes showed a decreased expression level during HF development, confirming their essential roles as master regulators during HF morphogenesis^10^. In addition to Wnt/beta-catenin related genes, genes in the TGF-beta/BMPs signaling pathway are also critical for HF morphogenesis, growth, and differentiation^8,54–56^. Consistent with these previous reports, the expression of TGF-beta/BMPs-related genes showed the highest level at either E55 or E65. *Shh*, *Notch1*, *Notch2*, and *Runx1* exhibited upregulation specifically at E65, supporting the finding that HF initiation might occur from E55 to E65.

Studies on cashmere goat embryonic HF have suggested considerable differences during physiological process at different stages of HF development. In this study, we reanalyzed the RNA-seq data from a previous study^23^ to compare the DEGs in E45/E55/E65 with the DEGs in E60/E120/NB (especially E120/NB) to further study the transcriptome dynamics from the early stage to the late stage of cashmere goat HF development. The early stage of HF development was only associated with PHF initiation while the later stage was associated with both PHF and SHF development^7^. Supporting the previous discovery, the global gene expression profile of E55 was different from that of E45 but similar with that of E65. From published data, the profile of E60 was different from that of both E120 and NB, while E120 and NB had similar gene expression profiles. The pathway analysis results of upregulated genes in specific stages were consistent with previous studies. Enriched functional pathways of genes in E60 and E65 were related to HF development. Pathways of genes upregulated in E60 and E65 were also different from those in E45, E120, and NB.

We also analyzed the expression patterns of key genes from canonical pathways in published RNA-seq data to explore their different functions between early and late stages of HF development. We took the Wnt/beta-catenin signaling pathway as an example, and saw that the expression profiles of *Wnt7a*, *APC*, *Wnt10a*, and *FZD8* showed similar patterns in the E45, E55, and E65 groups and E60, E120, and NB groups, while other genes showed different expression profiles in these two groups. From our results, we propose that these canonical pathways play different roles in the initiation or maturation of HF development. High expression of most genes from the WNT/β-catenin and NOTCH signaling pathways in the E65 and E60 samples implied their important regulatory role in HF initiation. Other factors that showed consistent increased expression levels during the course of embryonic development suggested that they have regulatory roles in both the initiation and development of HF. Other signaling pathways, such as TGF-β/BMP, FGF, and Hedgehog, had varied expression during development. Based on our analysis results, we extended the potential regulatory mechanisms of these signaling pathways during HF development. Further studies are necessary to explore their fundamental molecular mechanisms.

Because different embryonic stages play specific roles in cashmere goat HF development, transcriptional profiles have stage-specific features. This kind of stage-specific feature not only presented in the DEGs, but also in AS. It was clear that the RASEs could be divided into four groups, E55 specific, E65 specific, E45 specific, and E45&E55 specific, suggesting stage-specific features of AS events. Similar with the DEGs, AS-regulated genes between E45 and E55 were enriched for genes involved in the neurexins and neuroligins pathways, which are related to the neuronal system. Meanwhile, SHC-related events triggered by IGF1R and signaling by ERBB2 also emerged. AS-regulated genes in E65 vs E55 were enriched in pathways including cell-extracellular matrix interactions, smooth muscle contraction, and RHO GTPases. Although the functions of enriched pathways of AS genes and DEGs were similar, they may implement their functions through different pathways. From these results, we propose that AS regulation also influenced cashmere goat embryo HF initiation and development through pathways different from DEGs. HF initiation and development may be under the regulation of both transcription and post-transcriptional mechanisms.

In summary, we not only confirmed that the canonical signaling pathways and factors that influence human and mice HF development were highly regulated at the initiation stage of PHF (E45 to E65), but also showed that AS could play potentially important roles in embryonic HF initiation and development. Our study made a further step to understand the dynamic epithelial-mesenchymal interactions during cashmere HF initiation and development.

## Materials and Methods

### Ethics statement

The animal (including maternal goats and fetal goats) experiments were performed according to the Regulations for the Administration of Affairs Concerning Experimental Animals (Ministry of Science and Technology, China; revised in June 2004) and were approved by the ethics committee of Inner Mongolia Agricultural University. The animals did not experience unnecessary pain or distress at any stage of this experiment.

### Animal and skin tissue preparation

The Inner Mongolia cashmere goat is a traditional outstanding breed, which is famous for its excellent cashmere quality and strong adaptation to the semi-desert and desert pastures. The goats were grazed under the same environment with free access to feed and water at the Aerbasi White Cashmere Goat Breeding Farm (Inner Mongolia, China). Nine embryos (three samples at each stage) were randomly selected and any linage was avoided during the sampling process. The skin samples (approximately 1 cm^2^ for each individual) were collected from the mid flank region of embryos at three different embryonic days (E45, E55, E65). Tissue was frozen in liquid nitrogen and stored at −80 °C for further experiments.

### RNA extraction and RNA sequencing

Total RNA was extracted using TRIzol^®^ reagent (Invitrogen, Carlsbad, CA, USA) according to the manufacturer’s instructions, and RQ1 DNase (Promega, Madison, WI, USA) was used to remove contaminating genomic DNA. The quality and quantity of the purified RNA were monitored at the ratios of A260/A280 and A260/230 on a SmartSpecPlus Spectrophotometer (BioRad, Philadelphia, PA, USA). RNA integrity was further verified by 1.5% agarose gel electrophoresis.

For RNA-seq, total RNA was extracted from each embryo, and 10 mg of total RNA was used for RNA sequencing (RNA-seq) library preparation. Polyadenylated RNAs were purified and concentrated with Magnetic Beads Oligo (dT) (Invitrogen, Carlsbad, CA, USA) before being used for directional RNA-seq library preparation. Purified RNAs were iron fragmented at 95 °C followed by end repair and 5′ adaptor ligation. Then, reverse transcription was performed with RT primer harboring a 3′ adaptor sequence and randomized hexamer. The cDNAs were purified and amplified, and PCR products corresponding to 200–500 bps were purified, quantified, and applied to Illumina NextSeq. 500 system151nt pair-end sequencing by ABlife, Inc. (Wuhan, China).

### RT-qPCR experiments

To confirm the DEGs revealed by RNA-seq, six genes identified to be differentially expressed among three developmental stages were randomly chosen for reverse transcription qPCR (RT-qPCR) validation. Beta-actin was used as the internal control. The qPCR was carried out on an ABI 7300 system (ABI, USA) using FastStart Universal SYBR Green Master (ROX) according to the manufacturer’s instructions. The thermal cycling conditions used in the RT-qPCR reaction were 95 °C for 10 min, followed by 40 cycling of 95 °C for 10 s and 60 °C for 30 s. The specificity of the SYBR green PCR signal was confirmed by melting curve analysis. There were three biological and technical replicates. Primers for RT-qPCR are provided in Table S2.

### RNA-seq raw data cleaning and alignment statistics

Raw reads with more than 2-N bases were first discarded. Then, reads were processed by clipping adaptors and removing low quality bases, dropping too short reads (less than 16nt). FASTX-Toolkit (Version 0.0.13) was used to obtain the quality filtered reads. After that, the filtered reads were aligned to the goat (*Capra hircus*) genome^27^ by TopHat^57^. Based on the gene annotation of the genome, aligned reads with more than one genome location were discarded due to their ambiguous location. Uniquely localized reads were used to calculate read number and fragments per kilobase and per million (FPKM) value for each gene, according to the genomic locations of the aligned reads and genes. Other statistical results, such as gene coverage and depth and read distribution around start codons and stop codons, were also obtained.

### Differentially expressed genes (DEGs) analysis

We used RNASeqPowerCalculator^28^ to calculate the statistical power and other parameters for our RNA-seq data. Each sample was considered as a biological replicate. Differentially expressed genes (DEGs) between the three stages, E45, E55, and E65, were analyzed using the edgeR^29^ package in R software. For each gene, the false discovery rate (FDR) and log_2_ fold change (log_2_FC) were obtained based on the negative binomial distribution model. 0.05 FDR and 2-fold change were set as the thresholds to define DEGs.

### Alternative splicing analysis

The alternative splicing events (ASEs) and regulated alternative splicing events (RASEs) among these three developmental stages were defined and quantified using the ABLas pipeline as described previously^58^. In brief, detection of seven types of ASEs was based on the splice junction reads. The eight types of ASE included cassette exon (CassetteExon), exon skipping (ES), mutual exclusive exon skipping (MXE), A5SS, A3SS, the MXE combined with alternative 5′ promoter (5pMXE), and with alternative polyadenylation site (3pMXE). After detecting the ASEs in each RNA-seq sample, Fisher’s exact test was chosen to calculate the statistical *p*-value, with the alternative reads and model reads of samples as input data, respectively. The changed ratio of alternatively spliced reads and constitutively spliced reads between compared samples was defined as the RASE ratio. A *p*-value < 0.05 and RASE ratio > 0.2 were set as the thresholds for RASE definition.

### Functional enrichment analysis

Because goat genes lack functional annotation, we aligned the goat protein sequence to the sheep protein database by blastp^59^. The sheep protein with highest score was regarded as the homologous protein of the goat. Meanwhile, all differentially expressed genes were mapped to pathways in gene ontology (GO), Kyoto Encyclopedia of Genes and Genomes (KEGG), and Reactome databases. Fisher’s exact test was used to calculate the enrichment of each GO term and KEGG pathway. Reactome pathway analysis was performed online using Reactome version V62 (http://reactome.org/)^60^.

### Other statistical analysis

Hierarchical clustering was used to calculate the clustering of gene sets using Cluster3.0. In the heat map, the FPKM value of each gene was replaced by log_2_ (FPKM), the row-wise median was subtracted from the values in each row, then all values in each row were multiplied by a scale factor S so that the sum of the squares of the values in each row was 1.0. Other statistical results were obtained by R software.

## Supplementary information

Dataset 1

## Data Availability

The accession number for the RNA-seq data reported in this paper is GEO: GSE76538.
