# Supplementary material for: Transcriptome profiling reveals transcriptional and alternative splicing regulation in the early embryonic development of hair follicles in the cashmere goat
Source: Sci Rep. 2019 Nov 28;9:17735. doi: 10.1038/s41598-019-54315-7 (PMC6882815; doi:10.1038/s41598-019-54315-7)
Supplement: Supplementary file 1 — Dataset 1 [file 41598_2019_54315_MOESM1_ESM.docx]

**Transcriptome profiling reveals transcriptional and alternative splicing regulation in the early embryonic development of hair follicles in the cashmere goat**

Yanjun Zhang^1**^, Lele Wang^2**^, Zhen Li^3**^, Dong chen^3^, Wenjing Han^1^, Zhihong Wu^1^, Fangzheng Shang^1^, Erhan Hai^1^, Yaxun Wei^3^, Rui Su^1^, Zhihong Liu^1^, Ruijun Wang^1^, Zhiying Wang^1^, Yanhong Zhao^1^, Zhixin Wang^1^, Yi Zhang^3*^, Jinquan Li ^1,4,5,6*^

^1^College of Animal Science, Inner Mongolia Agricultural University, Hohhot, Inner Mongolia Autonomous Region, 010018, China.

^2^Key Laboratory of Animal Genetics, Breeding and Reproduction, Inner Mongolia Agricultural University, Hohhot, Inner Mongolia Autonomous Region, 010018, China.

^3^Center for Genome Analysis, ABLife, Inc., Wuhan, Hubei, 430075, China.

^4^Key Laboratory of Mutton Sheep Genetics and Breeding, Ministry of Agriculture ,Hohhot, Inner Mongolia Autonomous Region, 010018, China
^5^Key Laboratory of Animal Genetics, Breeding and Reproduction in Inner Mongolia Autonomous Region, Hohhot, Inner Mongolia Autonomous Region, 010018, China
^6^Engineering Research Center for Goat Genetics and Breeding, Inner Mongolia Autonomous Region, Hohhot, Inner Mongolia Autonomous Region, 010018, China

**These authors contributed equally to this work.

*Corresponding authors:

Jinquan Li

Key Laboratory of Animal Genetics, Breeding and Reproduction,

Inner Mongolia Agricultural University,

Hohhot, Inner Mongolia Autonomous Region, 010018, China

Tel: +86-13947131345

Fax: +86-471-4309170

E-mail: lijinquan_nd@126.com

Yi Zhang

ABLife, Inc.

Optics Valley International Biomedical Park, Building 9-4,

East Lake High-Tech Development Zone,

388 Gaoxin 2^nd^ Road,

Wuhan, Hubei 430075, China

Tel: 86-27-81779056

Email: yizhang@ablife.cc

**Table S1** Summary of RNA-seq data collected at the three early stages of prenatal hair follicles development.

| category | Early stages of prenatal hair follicles development (three replicates for each stage) | | | | | | | | |
| --- | --- | --- | --- | --- | --- | --- | --- | --- | --- |
|  | E45-1^a^ | E45-2 | E45-3 | E55-1 | E55-2 | E55-3 | E65-1 | E65-2 | E65-3 |
| raw reads | 69039998 | 74264218 | 69368776 | 76665994 | 76269258 | 76652558 | 66882470 | 95111216 | 74780136 |
| clean reads | 62650616 | 66754481 | 59033520 | 66480140 | 60836335 | 64438230 | 54093524 | 81195728 | 65382783 |
| aligned to  genome | 50812272  (83.61%)^b^ | 58847706  (85.55%) | 52603927  (84.0%) | 56247217  (84.51%) | 45398979  (81.8%) | 71694863  (85.44%) | 50931430  (83.04%) | 55373900  (84.67%) | 58368690  (86.32%) |
| uniquely  aligned | 44579727  (87.73%) | 51185008  (86.98%) | 45571213  (86.63%) | 48512265  (86.25%) | 39319085  (86.61%) | 62018283  (86.5%) | 43849186  (86.09%) | 46863883  (84.63%) | 50015915  (85.69%) |
| detected genes | 19701 | 20353 | 19996 | 19407 | 19868 | 19625 | 19217 | 19396 | 19432 |

^a^E, embryonic day.

^b^Read counts in the middle section are expressed in numbers (left) or as a percentage of the total reads processed (right) for each sample.

Table S2. Detail values of parameters obtained from statistical power analysis.

| **Parameters** | **Results** |
| --- | --- |
| Statistical power | 0.96 |
| RNA selection method | Poly-A enrichment |
| Experimental design | One factor |
| Number of samples | 9 |
| Median expression (log2 counts per million) | 5.24 (4.92-5.61) |
| Median log2-fold change of DE genes | 2.55 (1.15-17.47) |
| Median dispersion | 0.1368 (0.1352-0.2106) |
| Mean library size (sum of total counts, log10) | 7.74±.018 |
| Average sequencing depth (log10) | 7.815848 |
| Percent map to transcriptome | 84.32% |

Table S3. Primer list for RT-qPCR.

| **Gene** | **Forward primer (5’ to 3’)** | **Reverse primer (5’ to 3’)** |
| --- | --- | --- |
| *Smad1* | GATACACCCCCACCTGCTTA | CGCCTGAACATCTCCTCTGT |
| *Fgf10* | CGTCTCCTTGCCTTCCAGT | GCTGACCTTTCCGTTCTCAA |
| *Fgfr2* | GAACGATTACGGGTCCATCA | ACATCGCCTCCAACCACAG |
| *Wnt11* | GCGGCTACAACCCTTACACA | AAGTGGAGGCAAAGCACAAG |
| *Lef1* | ACCCATCCCAAGAACATCAA | CCTGGAGAAAAGTGCTCGTC |
| *Lrp6* | TTGCCTTAGATCCTTCAAGT | GGCCAGTAAATTTCAGTGTT |
| *β-actin* | GGCAGGTCATCACCATCGG | CGTGTTGGCGTAGAGGTCTTT |


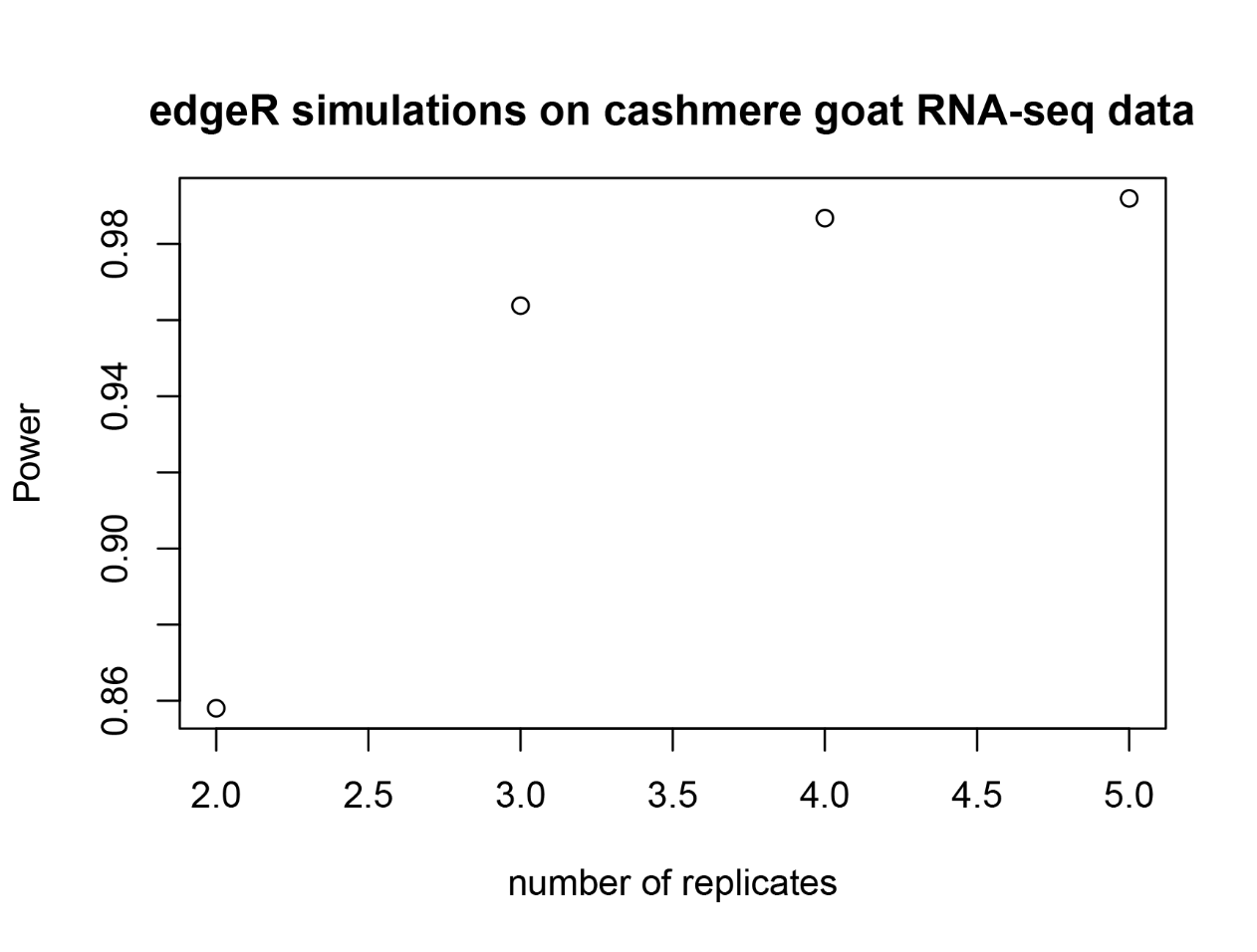


**Figure S1. Statistical power estimation presentation for our RNA-seq data using RNASeqPowerCalculator tool.**


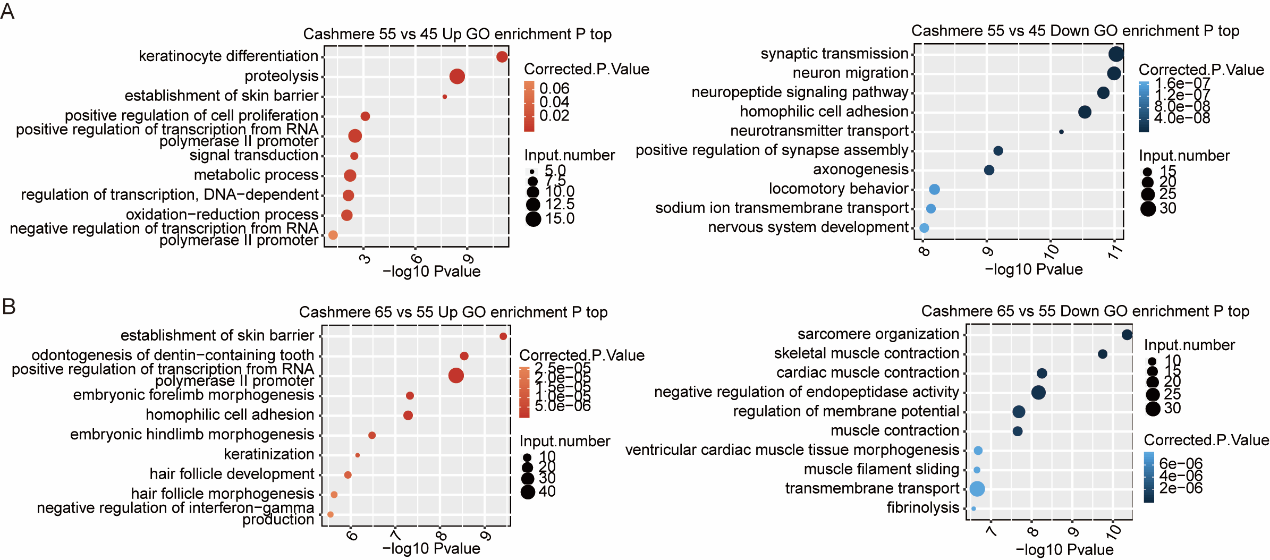


**Figure S2. The enriched GO biological process terms for the up-regulated (left panel) and down-regulated genes from E55 vs E45 (A) and E54 vs E55 (B) groups.**

**
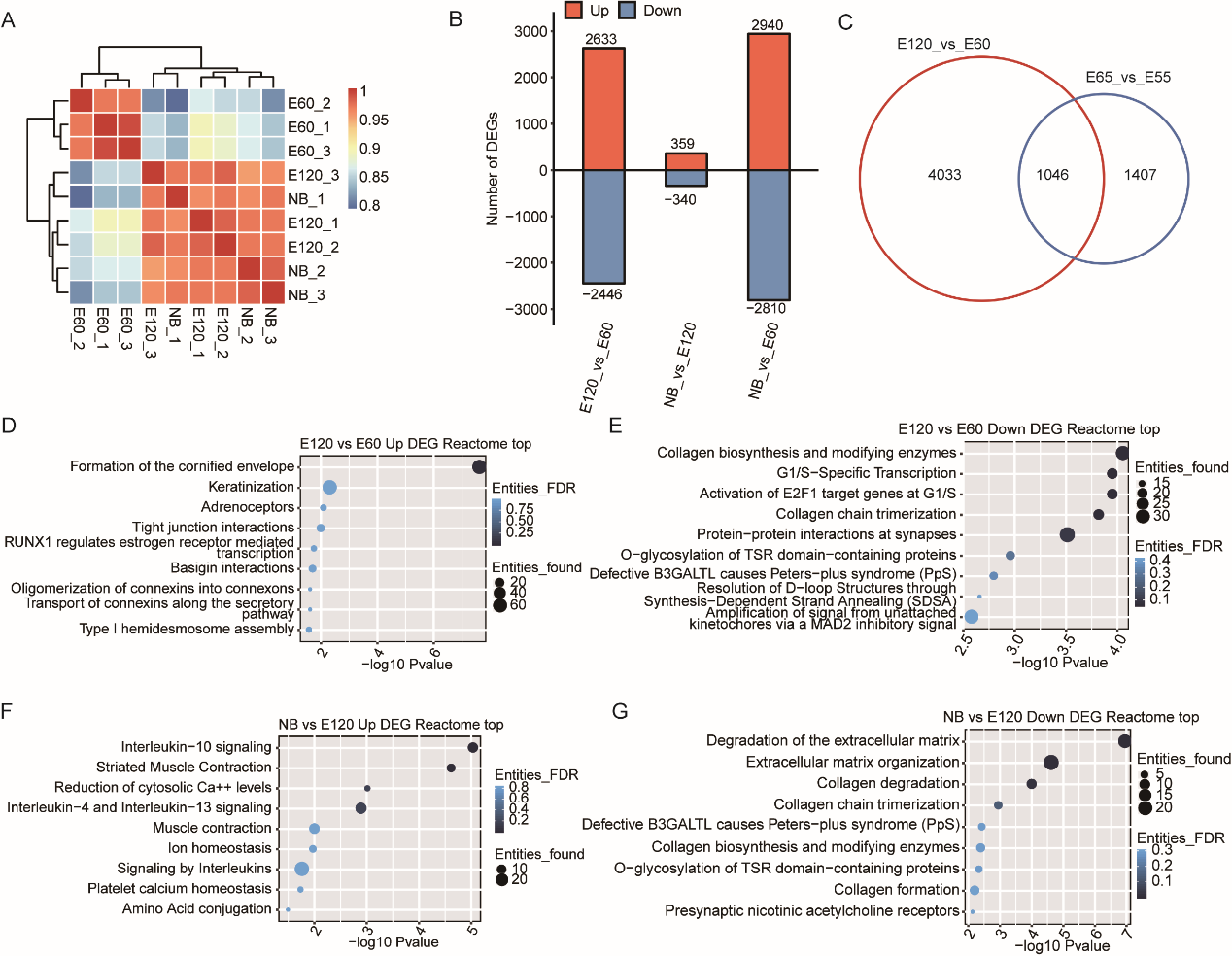
**

**Figure S3. The sample correlation and DEG analysis from published RNA-seq data. (A)** Heatmap showing the clustering of 9 embryonic samples by calculating correlation coefficients using expression values. **(B)** Barplot showing the number of differentially expressed genes in three comparing groups (E55 vs E45, E65 vs E55, E65 vs E45). Red bar emblems amount of up-regulated genes while blue emblems down-regulated genes. **(C)** Venn diagram showing the overlapped DEGs between E120 vs E60 and E65 vs E55. **(D-F)** Bubble plot showing the top enriched Reactome pathways of DEGs.
